# Isolation and Functional Analysis of a *PISTILLATA*-like MADS-Box Gene from Argan Tree (*Argania spinosa*)

**DOI:** 10.3390/plants10081665

**Published:** 2021-08-13

**Authors:** Marwa Louati, Blanca Salazar-Sarasua, Edelín Roque, José Pío Beltrán, Amel Salhi Hannachi, Concepción Gómez-Mena

**Affiliations:** 1 Faculty of Sciences of Tunis, Campus Farhat Hached El Manar, University of Tunis El Manar, Tunis 2092, Tunisia; marwa-louati@hotmail.com (M.L.); amel.hannachi@fst.utm.tn (A.S.H.); 2 Instituto de Biología Molecular y Celular de Plantas, Consejo Superior de Investigaciones Científicas, Universitat Politècnica de València, 46022 Valencia, Spain; blasasa@ibmcp.upv.es (B.S.-S.); edroque@ibmcp.upv.es (E.R.); jbeltran@ibmcp.upv.es (J.P.B.)

**Keywords:** *Argania spinosa*, Argan tree, *PISTILLATA*, flower, MADS-box gene, Tunisia

## Abstract

Argan trees (*Argania spinosa*) belong to a species native to southwestern Morocco, playing an important role in the environment and local economy. Argan oil extracted from kernels has a unique composition and properties. Argan trees were introduced in Tunisia, where hundreds of trees can be found nowadays. In this study, we examined reproductive development in Argan trees from four sites in Tunisia and carried out the functional characterization of a floral homeotic gene in this non-model species. Despite the importance of reproductive development, nothing is known about the genetic network controlling flower development in *Argania spinosa*. Results obtained in several plant species established that floral organ development is mostly controlled by MADS-box genes and, in particular, *APETALA3* (*AP3*) and *PISTILLATA* (*PI*) homologs are required for proper petal and stamen identity. Here, we describe the isolation and functional characterization of a MADS-box gene from *Argania spinosa*. Phylogenetic analyses showed strong homology with PI-like proteins, and the expression of the gene was found to be restricted to the second and third whorls. Functional homology with Arabidopsis *PI* was demonstrated by the ability of *AsPI* to confer petal and stamen identity when overexpressed in a *pi-1* mutant background. The identification and characterization of this gene support the strong conservation of *PI* homologs among distant angiosperm plants.

## 1. Introduction

*Argania spinosa* L. Skeels is an evergreen xerophyte, agroforestry species that belongs to the tropical family of Sapotaceae [1]. This species is known to be endemic to Morocco and Algeria [2]. Despite the rarity of this species outside of these two countries, Argan trees have been spotted in different stations in Tunisia. This can be explained by several introductory trials in a few stations in Tunisia between 1963 and 1968 [3]. However, long before these introductory attempts, Emberger (1925) [4] mentioned the existence of Argan trees in Tunisia, around Kairouan, a station not validated by botanists at the time [4]. Taking into consideration the geographical location of Tunisia, the hypothesis of the endemism of the Argan tree in Tunisia remains probable.

Since the past centuries, this species has proved to be essential for the economy of Morocco due to the production of Argan oil [5]. Moreover, the leaves and fruits of the Argan tree can be used as forage, and the plant is considered multipurpose, which increases its socioeconomic value [6]. Other than their considerable socioeconomic value, Argan trees have a great ecological interest. This species has no requirements for the type of soil and tolerates a wide pH range and high concentration of limestone [7]. Furthermore, Argan trees improve the quality of soil and water around them [8], and the root structures of this species and its high tolerance to aridity and high temperature make it a unique tool to counteract desertification [6]. The study of the argan tree population from Tunisia revealed large variability both within and between the studied sites [7]. The characterization and preservation of these populations could improve biodiversity in arid and semi-arid areas of Tunisia. In the long term, the effect on the ecosystem would be beneficial, and the establishment of the Argan tree can help the development of agriculture and the fight against wind erosion.

While many studies have been previously carried out to characterize the fruits, kernels, and leaves of the Argan [7,9,10,11], there is limited information concerning flower development [12,13,14,15]. Even more, to date, there is no available information of genes specifically involved in flower or fruit development in these species.

Flower development has been extensively studied in Arabidopsis and several model and non-model species, including crops. Genetics and molecular studies firmly established that floral organ development is genetically controlled by transcription factors encoded by genes from the MADS-box family [16,17]. Floral organs are arranged in concentric circles or whorls, and the number and disposition of the organs are characteristic of each species. MADS-box proteins interact in a combinatorial fashion to specify the identity of a particular floral organ. Thirty years ago, the ABC model was proposed and established three homeotic gene classes: A, B, and C [18]. Class A genes alone produce sepals, and in combination with B-class genes, regulate petal identity. Similarly, B and C function genes are required to establish stamen identity, and C functions alone to produce the carpel. Some years later, additional MADS-box genes were identified, resulting in two additional classes: D-class genes involved in carpel and ovule development [19,20] and E-class genes *SEPALLATA* genes that act redundantly in the control of all floral organs identities [21,22]. Altogether, the current model is known as the ABCDE model for flower development.

In Arabidopsis, petal and stamen development requires the presence of two B-class genes, *APETALA3* (*AP3*) and *PISTILLATA* (*PI*). Accordingly, mutations in either of these genes result in similar phenotypes with homeotic transformations of petals into sepals and stamens into carpels [23,24]. These two proteins, encoded by AP3 and PI, function as obligate heterodimers to bind DNA and directly regulate different sets of genes at distinct phases of flower development [25]. Several B-class gene orthologs have been identified and functionally analyzed in ferns, gymnosperms, and different types of angiosperms, and the biochemical aspects of AP3 and PI function appear to be conserved [26]. In the basal angiosperms, the study of AP3 and PI lineages shows dynamic patterns of gene lineage evolution with complex events of gene duplication [27]. It seems as though the evolution of the floral MADS-box gene, and in particular, AP3/PI lineages, is connected to the evolution of the flower itself and a source of major innovation during land plant evolution and angiosperm diversification [26].

Here, we studied reproductive development in the non-model species *Argania spinosa*. First, we examined the floral diversity of a collection of Argan trees from different geographical locations in Tunisia, and second, we isolated a *PI*-like B-class gene that we show is involved in petal and stamen identity. At present, there are no functional analyses of genes in Argan, mostly due to the absence of a reference genome publicly available. The molecular characterization of this gene and the generation of overexpression Arabidopsis lines provide the first functional analysis of a *PISTILLATA* MADS-box gene in this species.

## 2. Results

### 2.1. Argan Flower Anatomy and Development

**Figure 1:**
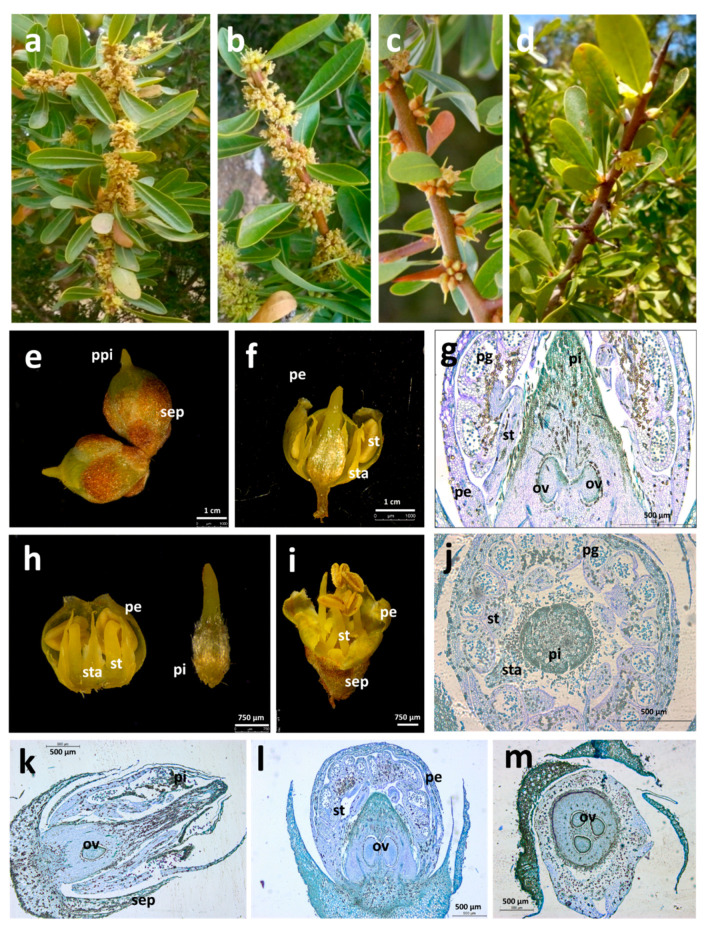
Reproductive development in *Argania spinosa* trees from Tunisia. Flower arrangements observed at the four sites: covering the whole shoot (**a**), helicoid (**b**), grouped as glomeruli in the axils of the leaves (**c**), and scattered (**d**). (**e**) Flower buds before anthesis showing a prominent pistil (ppi). (**f**,**h**,**i**) Dissected flowers showing Argan floral organs. (**g**,**k**,**l**) Longitudinal sections of flowers. (**j**,**m**) Transversal sections of flowers. Abbreviations: ov (ovule), pe (petals), pg (pollen grains), pi (pistil), ppi (protrudent pistil), sep (sepals), st (stamen), sta (staminoids).

We took advantage of the available Argan population in Tunisia to study the diversity of flower disposition and number. Characterizing flower number variability in Argan populations could be interesting for the selection of desirable genotypes for future breeding programs in Tunisia. Observation of Argan trees at the four stations revealed a very high level of phenotypic variability during reproductive development. Four types of flower arrangements were observed: covering the whole shoot, helicoid, grouped as glomeruli in the axils of the leaves, and scattered (Figure 1a–d). The flowers appear on the axils of leaves or spines or on the nodes of mature wood. Argan tree is a monoecious species, with zygomorphic, bisexual, pentameric hermaphrodite flowers that have a pleasant smell [14]. The outermost whorls are composed of five hairy, rounded brownish sepals and five rounded greenish-yellow or white petals (Figure 1). The androecium is composed of five stamens forming a ring around the carpel that protrudes before flower opening (Figure 1e). Dissection of Argan flowers showed that the petals are attached to each other and to the stamens at the base and revealed the presence of five short staminodes (Figure 1f,h). Histological sections of flowers from different sites showed pistils with a single ovule, two ovules, or even three ovules (Figure 1j–m). The most frequent number of ovules per flower was two (average number of ovule per flower = 1.9 for botanical garden; 2.27 for Korbous; 1.66 for Sousse and 2.21 for Sfax) independently of the population analyzed.

In contrast, the four stations showed variability in terms of the number of flowers per tree and the disposition of flowers in the branches (Table 1). Regarding flower frequency per branch, we considered four phenotypical classes: high (>150), medium (50–150), low (1–50), and none (0). The most frequent number of flowers per branch in the entire tree population was medium (50–150 flowers/branch) and also in the individual sites. The exception was the trees from the Korbous site that showed more than 150 flowers/branch (Table 1). Regarding flower disposition, 43% of the trees analyzed showed flowers grouped as glomeruli, although we observed differences in flowers disposition inter and intra population. Helicoidal disposition of flowers is more frequent in trees from the Botanic Garden and Korbous. Remarkably, the percentage of scattered single flowers is higher in Sfax and Sousse than in Korbous and the botanical garden. The presence of flowers covering the whole shoot was infrequent (Table 1).

### 2.2. Cloning and Sequence Analysis of a PISTILLATA Gene from Argania spinosa

To identify the *PISTILLATA* homolog in *Argania spinosa*, we searched DNA databases for PI coding sequences from trees or woody plants. We selected a subset of seven *PI*-like sequences previously characterized as B-function genes [28,29,30,31,32]. The alignment of the sequences identified a fragment over 300 bp highly conserved among all sequences starting from the start codon and another shorter but similarly conserved region near the C-terminal end (Supplementary Figure S1). We then designed degenerated primers to amplify the first region, both primers containing variations in four of the positions (Supplementary Table S1). Using these primers and cDNA obtained from Argan flowers, we amplified and cloned a DNA fragment corresponding to a partial coding sequence of the *AsPI* (*Argania spinosa PISTILLATA*) gene.

By means of 5′ and 3′ RACE experiments, we obtained the full coding sequence of the *AsPI* gene (GenBank MW650858) that encoded a deduced protein of 208 aa (Supplementary Figure S2a). The predicted amino acid sequence contains three conserved regions annotated as MADS-box domain (1–61 aa), I-box (62–83 aa) and K-box domain (84–170 aa), and the consensus PI-motif (MPFxFRVQPxQPNLQE) in the C-terminal region (Supplementary Figure S3), which is specifically found in PI orthologs [33]. Additionally, we obtained a collection of sequences of variable length corresponding to the 3′UTR region (Supplementary Figure S2b). Arabidopsis and Argan PI proteins showed 62.0% total amino acid identity (85.6% similarity).

**Figure 2:**
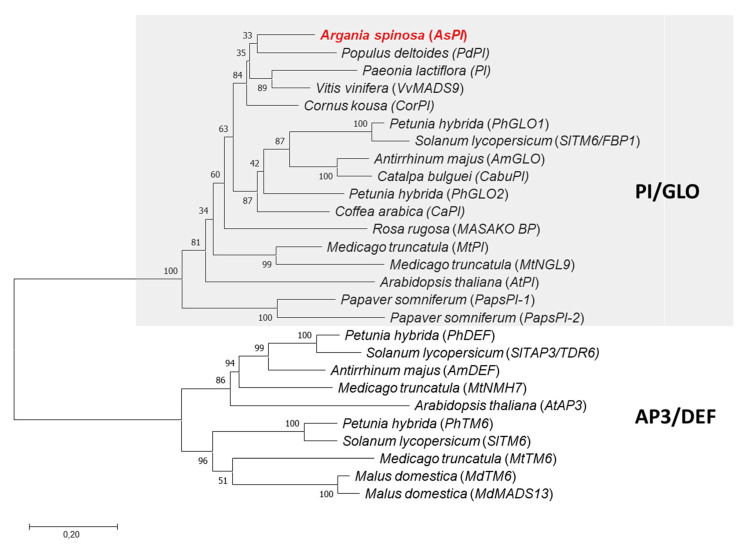
AsPI is a PISTILLATA-like MADS-box protein. Phylogenetic reconstruction using the maximum likelihood (ML) method based on PI/GLO- and AP3/DEF-related MADS-box proteins from different species. The PI/GLO clade has been highlighted with a gray square. *Argania spinosa* PI (AsPI) protein is labeled in red. The number close to the nodes indicates the bootstrap support values from 10,000 pseudo-replicates. The accession numbers of the sequence data used are listed in Supplementary Table S2.

We used the coding sequence of the *PI* gene from Argan to perform phylogenetic analysis with AP3 and PI proteins from different plants species. We included functionally characterized PI proteins from distant phylogenetic plants. The topology of the tree showed that the *AsPI* gene is closely related to members of the PI/GLO subfamily (Figure 2). *AsPI* falls within the well-supported PI/GLO clade (bootstrap value of 100%). This clade is well defined and separated from the AP3/DEF clade (Figure 2).

### 2.3. Expression Profiling of AsPI in Argan Flowers and Leaves

**Figure 3:**
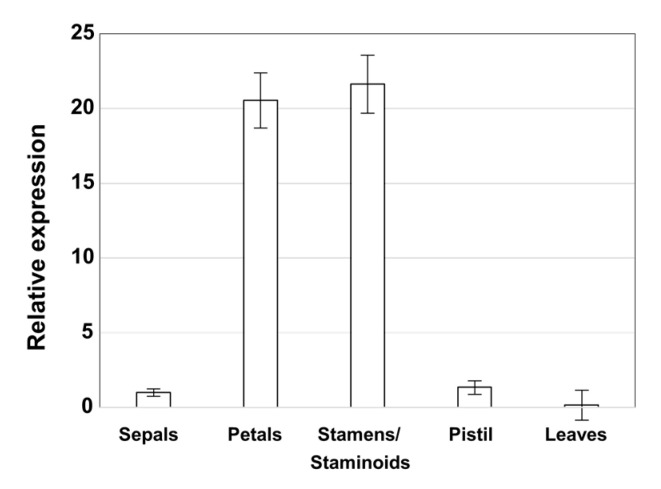
Relative expression of *AsPI* in leaves and flower organs analyzed by qRT-PCR. Data were normalized to the expression of the *ycf2* gene and correspond to the mean (±SD) of three replicates.

MADS-box genes involved in floral organ identity are expressed in distinct floral whorls. Class A genes are expressed in developing organ primordia of sepals and petals, class B genes in petals and stamens, and class C genes in stamens and carpels. Therefore, we analyzed the expression pattern of *AsPI* in different floral organs and leaves by quantitative RT-PCR. In agreement with the expected expression pattern for a B-function gene, *AsPI* was expressed in petals and stamens/staminoids and barely detectable in sepals and carpels, and was not expressed in leaves (Figure 3), showing that *AsPI* expression is flower-specific. In summary, *AsPI* sequence analyses and the expression pattern of the gene correspond to a typical *PISTILLATA* MADS-box B-class gene.

### 2.4. Subcellular Location of AsPI Protein in Nicotiana benthamiana

**Figure 4:**
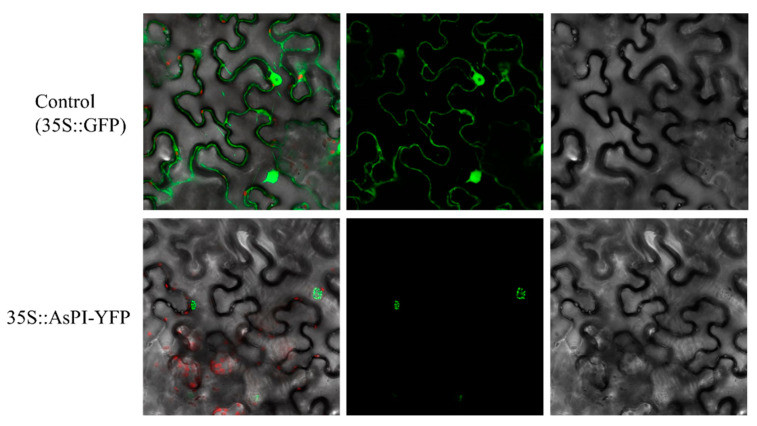
Subcellular localization of AsPI protein in *Nicotiana benthamiana* leaves as observed by confocal microscopy. Upper panel: cytoplasmic and nuclear localization of the 35S::GFP control construct. Lower panel: nuclear localization of the 35S::AsPI-YFP construct. From right to left, the three independent images correspond to bright field, GFP spectrum, and the overlay of both images.

Since PI proteins are transcription factors, they are expected to be localized into the nucleus. To investigate the subcellular localization of the AsPI protein, we produced a translational fusion of AsPI with the YFP fluorescent protein. The expression of the fusion protein was driven by a constitutive 35S promoter and transiently expressed in epidermal cells of *N. benthamiana* leaves (Figure 4). Plants expressing the 35S::GFP control showed strong fluorescence in the cytoplasm and nucleus. In contrast, we observed intense fluorescence of the AsPI-YFP protein exclusively in the nuclei (Figure 4). The analysis *in silico* of the protein indicated that the AsPI protein carries potential nuclear localization signals in its N-terminus (Supplementary Figure S2a).

### 2.5. AsPI Overexpression in Arabidopsis and pi-1 Mutant Complementation

To analyze the function of the isolated *AsPI*, we overexpressed the gene in the model plant Arabidopsis. We also tested whether AsPI could replace AtPI function in Arabidopsis by overexpression of the Argan gene in the *pi-1* mutant background, a loss-of-function mutant allele with a strong floral phenotype [24,34].

**Figure 5:**
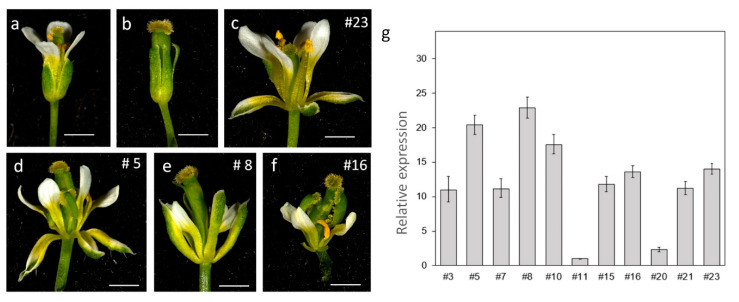
Overexpression of *AsPI* in Arabidopsis plants and complementation of *pi*-1 floral phenotype. (**a**) Wild-type flower; (**b**) *pi*-1 flower; (**c**) Flower from 35S::*AsPI* transgenic plants (line #23); (**d–f**) Flowers from *pi*-1; 35S::*AsPI* plants (lines #5, #8 and #16) showing different degrees of complementation. (**g**) Relative expression of *AsPI* gene in the Arabidopsis 35S::*AsPI* transgenic lines. All the transgenic lines are homozygous for the *pi*-1 mutation except for line #23 that does not contain the mutation. Data were normalized to the expression of the *AtTIP41* gene, and the gene expression level in line #11 was set as 1. Each value corresponds to the mean (±SD) of three replicates.

We transformed a 35S::*AsPI* construct into heterozygous *pi-1* plants. Twenty-four transformants were recovered in plates containing selection antibiotics and genotyped for the presence of the *pi-1* mutation. A total of 12 of the transgenic lines were homozygous for the mutation, 10 were heterozygous, and 2 did not contain the mutation (Supplementary Figure S4). The expression of the transgene was analyzed in 11 lines (Figure 5g), of which 10 were homozygous for the *pi-1* mutation and 1 (35S::*AsPI* #23) that did not contain the mutation.

The ectopic expression of *AsPI* in a wild-type background modified flower anatomy as expected. We observed previously described phenotypes caused by overexpression of the Arabidopsis *PI* [35] and *PI* homologs [29,36], including strong separation between sepals, sepals, and petals narrowing and sepals edge whitening (Figure 5C). The white areas in sepals are attributed to the partial homeotic transformation of sepals into petals [29,35,36]. Our results indicate that *AsPI* is able to induce B-function identity when ectopically expressed in Arabidopsis plants. In addition, we analyzed the floral phenotypes of the overexpressing lines in the *pi-1* background. The flowers of the *pi-1* mutant contain a first outer whorl of sepals, a second whorl of sepal-like organs, and an abnormally large gynoecium in the center of the flower. Stamens are missing, and, occasionally, filamentous structures appear [34,37] (Figure 5 and Table 2).

Among the overexpressing lines, we observed a low level of complementation in two of the lines (*pi-1*, 35S*::AsPI* #11 and *pi-1*, 35S*::AsPI* #20; Table 2) that correlate with the low expression level of *AsPI* detected in these plants (Figure 5g). The remaining lines showed full recovery of petals in the second whorl and partial recovery of staminoid structures in the third whorl (Figure 5 and Table 2). In the first whorl, we observed the characteristic white sepal edges that correspond to petaloid tissues, but the more conspicuous phenotype was the full recovery of white petals (Figure 5d–f). In the third whorl, we recorded an increase in the number of organs compared to the *pi-1* mutant (Table 2). These organs correspond to filaments (92% of the flowers analyzed/line), filaments ending in stigmatic tissue (20%–30% of the flowers analyzed/line), and occasionally, filaments with anther tissue (10%–20% of the flowers analyzed/line) (Figure 5d–f and Table 2). In the innermost whorl, we observed many carpel abnormalities: opened, curved or incomplete carpels (Figure 5d–f) caused by the ectopic expression of *AsPI* in this tissue. In summary, the observed complementation of the *pi-1* floral phenotype by *AsPI* confirms its ability to replace PI function.

## 3. Discussion

The analyses of the flower disposition and flower number of the argan tree population for Tunisia showed an important variability within and between sites. This could be the consequence of influence at different levels: geographic origin, genotype (tree/location), and genotype x environment interaction. The flowering-fruiting cycle covers a period of 9 to 16 months for the Argan trees in Morocco, from flower opening to fruit maturation [12]. Season was the main source of variation in the number of glomeruli on Argan shoots, which may explain conflicting reports on flowering times in the literature [36]. Other studies associate the precocity of flowering with the precocity of precipitation [15,38]. Argan populations from sites characterized by a drier and hotter climate (Sfax) showed the lowest frequency of flower per branch and often as scattered single flowers. However, in the areas characterized by higher pluviometry (botanical garden and Korbous), we recorded the highest frequency of flowers. Besides the differences, we observed higher homogeneity among trees from Korbous, Sousse, and Sfax sites than within the botanical garden population. In addition, trees from Sousse and Sfax seem to be more closely related, in agreement with previous analyses [7,11].

In this study, we used a molecular approach to identify regulatory genes involved in floral organ development in Argan. We isolated and functionally characterized a MADS-box gene from *Argania spinosa*. Phylogenetic analysis showed that *AsPI* falls within a clade that includes several *PI/GLO*-like genes. In this group, Arabidopsis *PI* and *Antirrhinum majus GLO* (*AmGLO*) correspond to single genes while the petunia and Medicago counterparts correspond to duplicated genes, *PhGLO1/PhGLO2* and *MtPI/MtNGL9* in petunia and Medicago, respectively [29,33,39,40,41,42,43]. Gene duplications in the *GLO/PI* lineage have led to functional divergence and specialization of these MADS-box genes [29,41,44]. B-function MADS-box genes show multiple examples of gene duplication followed by gene loss or paralogs diversification, suggesting that gene duplication has been a crucial factor in shaping flower evolution [45,46]. Our analysis identified a single *PI* copy in the Argan genome, but we cannot fully exclude the presence of additional gene copies. Further analyses will be needed to support or discard the presence of duplicated genes in the *PI/GLO*-like lineage in this species.

Floral MADS proteins share a common modular conserved structure with four characteristic domains: MADS domain (M), intervening domain (I), keratin-like domain (K), and C-terminal (C) domains [47]. At the C-terminal region, most PI lineage proteins, including AsPI, harbored a highly conserved sequence of approximately 16-amino acid (PI-motif) considered essential for protein function [48]. However, additional research demonstrated that C-terminal motifs of AP3 and PI proteins are not required for floral organ identity specification [41,49,50]. The *AsPI* transcript, isolated from Argan flowers, encodes for a typical PI protein showing the classical MIKC structure and the presence of the conserved PI-motif. In lily (*Lilium longiflorum*), two *PI* genes were characterized, one of them lacking the PI-motif, and it was found that its presence was related to the ability of PI proteins to form homodimers [51].

*AP3* and *PI* genes are present as a single copy in the Arabidopsis genome and are required for the specification of petals and stamens. Although their pattern of expression during flower development are similar, their single ectopic expression causes different phenotypic alterations. Overexpression of *PI* causes partial first whorl conversion of sepals to petals [37], whereas plants ectopically expressing *AP3* exhibit partial conversion of carpels to stamens [52]. Simultaneous ectopic expression of both genes causes vegetative phenotypes (curling of leaves) and early flowering. In these plants, the flowers show stronger homeotic transformation on the first and fourth whorls; sepals tissues are absent on the outermost whorl, and carpelloid structures are rarely found in the innermost whorl [37].

Ectopic expression of *AsPI* in Arabidopsis wild-type plants phenocopies the effects described for the Arabidopsis *PI* gene. The flowers showed partial transformation of sepals into petaloid organs. In addition, sepals are widely separated in 35S*::AsPI* transgenic flowers, a phenotype associated with the ectopic expression in Arabidopsis of several *PI* orthologs from distant plant species such as *Medicago truncatula* [29], *Catalpa bungei* [35], *Lilium longiflorum* [51], among others. Interestingly, the ectopic expression of *TrPI*, a *PI* ortholog from the eudicot species *Taihangia rupestris* (Rosaceae) in Arabidopsis caused severe modifications in vegetative plant architecture [53]. Besides specifying the identities of petals and stamens, *TrPI* might function in regulating plant architecture in accordance with its expression in leaves and inflorescence stems [53]. We did not observe defects in vegetative development because of the ectopic expression of *AsPI,* suggesting a specific role for this gene during Argan flower development.

Previous studies in Arabidopsis showed that the simultaneous overexpression of *PI* and *AP3* in different *pi* mutant alleles have different degrees of complementation of the mutant phenotype. Ectopic expression of *PI* and *AP3* fully rescues a *pi-2* mutant but only partially *pi-1* or *pi-3* mutant alleles that showed full recovery of petals identity but only mild recovery of stamens in addition to carpel defects [37]. The *pi-1* allele was obtained by EMS (ethyl methanesulfonate) mutagenesis and contained a point mutation that introduces a premature stop codon (TGG to TGA). The mutation results in a 79aa truncated protein and are considered a null allele [24]. We obtained similar results overexpressing *AsPI* in the *pi-1* mutant background, confirming the ability of *AsPI* to confer B-class organ identity function in the *Arabidopsis* heterologous system.

The isolation and functional characterization of the *AsPI* gene is a pioneering study on the understanding of the molecular network controlling flower development in *Argania spinosa*. Our results support the functional conservation of MADS-box factors regulating the formation of the floral organs in angiosperms. Moreover, these findings provide molecular information for future studies in this species, a non-model plant with an ecologic value and local economic importance.

## 4. Materials and Methods

### 4.1. Plant Material

*Argania spinosa* flowers were collected from 60 Argan trees located in four stations in Tunisia: Tunis Botanical Garden, Korbous, Sousse, and Sfax (Table 3). The four locations have differences in altitude, climate, and soil composition [7]. The Argan trees located in Tunis at the botanical garden (Site 1) were introduced decades ago from Morocco. The rest of the sites contained trees from an unknown origin.

Arabidopsis plants were grown in growth chambers at 21 °C under long-day (16 h light/8 dark) conditions, in a mixture of 1:1:1 soil:perlite:vermiculite. Wild-type *Arabidopsis thaliana* seedling (Landsberg *erecta* accession) and *pistillata-1* (*pi-1*) mutant [24,48].

### 4.2. RNA Extraction and First-Strand cDNA Synthesis

Total RNA was isolated from Argan flower buds using RiboZol RNA extraction reagent (VWR Life Science, Solon, OH, USA) following the manufacturer’s instructions. After the extraction, we treated the samples with DNAase (Turbo DNA-free kit; Invitrogen, Waltham, MA, USA) to eliminate possible contamination with traces of genomic DNA. First-strand cDNA synthesis was performed by reverse transcription using 1 µg of total RNA and with the system Primer Script RT reagent kit (TaKaRa, Shiga, Japan) using a mix of poly-dT and random primers.

### 4.3. Cloning of AsPISTILLATA

A partial sequence of the *AsPI* gene was isolated from first-strand cDNA obtained from flower buds and using degenerated oligos PI-DEG For and PI-DEG Rev. The PCR product (321 bp) was purified and inserted into the pGEM-T Easy vector (Promega, Madison, WI, USA) for sequencing. The cloned partial cDNA sequence was used to design gene-specific primers to amplify the 5′ and 3′ end of cDNA using the SMARTer RACE 5′/3′ Kit (TaKaRa Bio, Shiga, Japan) according to the manufacturer’s instruction. The gene-specific primers were AsPI GSP2 for 3′ RACE and AsPI GSP7 for 5′ RACE. The sequence of the primers used is listed in Supplementary Table S1.

### 4.4. Phylogenetic Analyses

The evolutionary history was inferred by using the maximum likelihood method and Tamura-Nei model [54]. The tree with the highest log likelihood (−13,496.01) is shown. Initial tree(s) for the heuristic search were obtained automatically by applying Neighbor-Join and BioNJ algorithms to a matrix of pairwise distances estimated using the Tamura-Nei model and then selecting the topology with a superior log likelihood value. A discrete Gamma distribution was used to model evolutionary rate differences among sites (5 categories (+G, parameter = 1.8084)). The rate variation model allowed for some sites to be evolutionarily invariable ([+I], 9.65% sites). The tree is drawn to scale, with branch lengths measured in the number of substitutions per site. The analysis involved 26 DNA sequences obtained from GenBank and the *PI-like* sequence that we isolated from *A. spinosa*. Codon positions included were 1st + 2nd + 3rd + Noncoding. There were a total of 803 positions in the final data set. Evolutionary analyses were conducted in MEGA X [55]. Sequences used in this analysis are listed in Supplementary Table S2.

### 4.5. Expression Analyses

For expression analyses, leaves and flower buds were collected. Flower buds were dissected under a stereomicroscope (Nikon, Tokyo, Japan) to separate floral worlds (sepals, petals, stamens/staminoids, and pistil) before total RNA extraction. First-strand cDNA synthesis was performed as previously described. The cDNA was diluted 10-fold with RNAase-free water for qRT-PCR. Quantitative RT-PCR (qRT-PCR) was carried out with cDNA and Pyro Taq EvaGreen qPCR Mix (Cultek) using the QuantStudio 3 Real-Time PCR System (Applied Biosystems, Waltham, MA, USA). In a single experiment, each sample was assayed in triplicate (technical replicates).

For the expression analyses in Argan, the *ycf2* gene (GenBank: JQ290464) was chosen as the internal control. For expression analyses in Arabidopsis, we used the *TIP4* gene (*At4g34270*) as the internal control. The amplification efficiency of each set of primers was tested before the expression studies. Relative expression levels were calculated using the endogenous control genes as normalizers with the 2^−ΔΔCt^ method [56].

### 4.6. Subcellular Localization of AsPI Protein by Transient Expression in Nicotiana benthamiana

The complete *AsPI* cDNA was amplified using AsPI ATG For and AsPI Rev primers (Primers are listed in Supplementary Table S1) and cloned by recombination into the pEarleyGate 101 vector [57] to obtain an in-frame fusion to the fluorescent YFP protein. The final construct was confirmed by sequencing. Constructs containing AsPI-YFP fusion proteins were transformed into *Agrobacterium tumefaciens* C58/pMP90.

Overnight cultures of Agrobacterium containing the construct were diluted in infiltration buffer and used to infiltrate 4-week-old *Nicotiana benthamiana* leaves [58]. After 48 h of the infiltration, the localization of the fluorescent protein was determined on leave disks by confocal scanning microscopy (LSM 780, Zeiss, Jena, Germany) analyses.

### 4.7. Arabidopsis Transformation for AsPI Overexpression

For the overexpression of the AsPI protein, the coding sequence of the gene was cloned by recombination into de pEarleyGate100 vector [57] that placed the cDNA under the cauliflower mosaic virus 35S promoter. The final construct was confirmed by sequencing.

Arabidopsis plants heterozygous for the *pi-1* mutation were transformed by floral dipping according to standard procedures [59] after electrophorating the generated plasmid (35S::*AsPI*) into Agrobacterium strain C58/pMP90. Transformants were selected on phosphinotricin (PPT; Duchefa Biochemie, Haarlem, The Netherlands).

### 4.8. Histological Studies of Argan Flowers

For histological analyses, flower buds at different developmental stages were taken and fixed in FAE solution (3.7% formaldehyde, 5% acetic acid, 50% EtOH) overnight at 4 °C. The next day, FAE solution was removed and samples dehydrated in increasing concentrations of ethanol (70%, 80%, 90%, 95%, and 100%), cleared with Histo-Clear (VWR Life Science, Solon, OH, USA), and embedded in paraffin. Microtome sections of 8 µm were obtained and stained with 0.05% toluidine blue [60]. Stained slides were examined under a bright field using a Leica DM5000 light microscope equipped with a digital camera.

## Figures and Tables

**Table 1:** Flowering phenology among trees from the four sites. Number of trees analyzed per site *n* = 15. The most frequent classes for every site and for the entire population are indicated in bold.

Site	Flower Disposition (% of Trees)				Flower Frequency/Branch (% of Trees)			
	Covering the Whole Shoot	Helicoidal Disposition	Grouped as Glomeruli	Scattered	>150	50–150	1–50	0
Botanical Garden (BG)	13.3	33.3	40.0	13.3	33.3	53.3	6.7	6.7
Korbous (A)	13.3	20.0	46.7	20.0	60.0	26.7	13.3	6.7
Sousse (H)	0.0	13.3	46.7	40.0	20.0	53.3	20.0	6.7
Sfax (S)	6.7	0.0	40.0	53.3	20.0	33.3	33.3	13.3
All trees	8.3	16.7	43.3	31.7	33.3	41.7	18.3	8.3

**Table 2:** Complementation of *pi-1* floral phenotype by *AsPI* overexpression. Average number of organs per whorl and phenotypes observed (*n* = 10–20).

Genotype	1st Whorl	2nd Whorl	3rd Whorl	4th Whorl
Wild-type	4.00 ± 0,00	4.00 ± 0.00	5.82 ± 0.50	2.00 ± 0.00
*pi-1* mutant	4.00 ± 0,00	4.00 ± 0.00 ^b^	0.19 ± 0.40 ^c^	2.76 ± 0.54 ^g^
*pi-1,* 35S::*AsPI* #3	4.00 ± 0,00 ^a^	4.00 ± 0,00	5.10 ± 0.88 ^c,d^	2.30 ± 0,67 ^g^
*pi-1,* 35S::*AsPI* #5	4.00 ± 0,00 ^a^	4.00 ± 0,00	5.00 ± 0.82 ^c,d,e^	2.30 ± 0.48 ^g^
*pi-1,* 35S::*AsPI* #7	4.00 ± 0,00 ^a^	4.00 ± 0,00	4.24 ± 1.60 ^c,d,e,f^	2.12 ± 0.33 ^g^
*pi-1,* 35S::*AsPI* #8	4.00 ± 0,00 ^a^	4.00 ± 0,00	2.57 ± 1.29 ^c,d,e,f^	2.62 ± 0.59 ^g^
*pi-1,* 35S::*AsPI* #10	4.00 ± 0,00 ^a^	4.00 ± 0,00	3.60 ± 1.64 ^c,d,e,f^	2.10 ± 0.31 ^g^
*pi-1,* 35S::*AsPI* #11	4.00 ± 0,00 ^a^	4.00 ± 0,00 ^b^	1.00 ± 1.00 ^c^	3.00 ± 0.00 ^g^
*pi-1,* 35S::*AsPI* #15	4.00 ± 0,00 ^a^	4.00 ± 0,00	4.15 ± 1.31 ^c,d^	2.30 ± 0.47 ^g^
*pi-1,* 35S::*AsPI* #16	4.00 ± 0,00 ^a^	4.00 ± 0,00	4.60 ± 0.70 ^c,d,e,f^	2.40 ± 0.52 ^g^
*pi-1,* 35S::*AsPI* #20	4.00 ± 0,00	4.00 ± 0.00 ^b^	0.55 ± 0.83 ^c^	2.45 ± 0.51 ^g^
*pi-1,* 35S::*AsPI* #21	4.00 ± 0,00 ^a^	4.00 ± 0,00	3.60 ± 1.96 ^c,d^	2.90 ± 0.99 ^g^

In the wild-type, floral worlds correspond to sepals, petals, stamens, and carpels. Novel phenotypes observed that differ from morphological wild-type organs: ^a^ white sepal edges; ^b^ sepal-like organs; ^c^ filaments; ^d^ filaments fused to carpel; ^e^ filaments with stigmatic tissue; ^f^ anther tissue; ^g^ incomplete, bent or abnormal carpel.

**Table 3:** Localization and general climatic conditions of the sites of the collection of Argan trees used in this study.

Site Name and Code	Individual Code	Geographic Region	Bioclimatic Stage	Latitude	Longitude	Rainfall (mm/year)
Botanical Garden (BG)	P1A, P2F1, P3A2, P3A6, P3B8, P3C3, P4A8, P5F9, P6H9, P8G10, P9A11, A1JB, A3JB, A6JB, A8JB	North	Upper semi-arid	36°81′ N	10°16′ E	400–500
Korbous (A)	A1, A2, A3, A4, A5, A6, A7, A8, A9, A10, A11, A12, A13, A14, A15	Northeast (Cap Bon)	Sub-humid	36°81′ N	10°56′ E	500–700
Sousse (H)	H1, H2, H3, H4, H5, H6, H7, H8, H9, H10, H11, H12, H13, H14, H15	Central-East (Sahel)	Lower semi-Arid	35°82′ N	10°64′ E	300–400
Sfax (S)	S1, S2, S3, S4, S5, S6, S7, S8, S9, S10, S11, S12, S13, S14, S15	Southeast	Upper arid	34°72′ N	10°33′ E	100–200

## Data Availability

All data are included within the article.

## Supplementary Materials

The following are available online at https://www.mdpi.com/article/10.3390/plants10081665/s1, Figure S1. Alignment of partial cDNA sequences (360 nucleotides) of *PISTILLATA* genes from selected woody species and from the mode legume *Medicago truncatula*. Figure S2. *PISTILLATA* gene sequence. Figure S3. Protein alignment of AsPI and functional characterized PI/GLO-related proteins from different species. Figure S4: Genotyping of transgenic plants transformed with 35S::*AsPI* for the presence of the *pi-1* mutation. Table S1: Primers used in this work. Table S2: Accession numbers of the *AP3/DEF*- and *PI/GLO*-related MADS-box genes used for phylogenetic analysis.
