# Supplementary material for: Isolation and Functional Analysis of a PISTILLATA-like MADS-Box Gene from Argan Tree (Argania spinosa)
Source: Plants (Basel). 2021 Aug 13;10(8):1665. doi: 10.3390/plants10081665 (PMC8399449; doi:10.3390/plants10081665)
Supplement: Supplementary file 1 [file plants-10-01665-s001.zip › plants-1309658-supplementary.pdf]

```
Mt      ATGGGAAGAGGTAAGATTGAGATCAAGAGGATTGAAAACTCAAGCAACAGGCAAGTTACT      60
Cb      ATGGGTAGAGGCAAGATTGAGATCAAGAGAATCGAGAACTCAAGCAACAGACAGGTTACT      60
Ca      ATGGGGAGAGGTAAGATTGAGATCAAGAGGATTGAGAACACAAACAACAGGCATGTGACT      60
Pl      ATGGGAAGAGGTAAGATTGAGATCAAGAGGATTGAGAACTCAAATAACAGGCAGGTGACC      60
Pd      ATGGGGAGAGGTAAGATTGAGATCAAGAGGATTGAGAACGCAAGCAACAGGCAGGTGACC      60
Ck      ATGGGGAGAGGAAAGATAGAGATAAAGAGGATAGAGAACTCAAGCAACAGGCAGGTCACA      60
Vv      ATGGGGAGAGGGAAGATTGAGATCAAGAGGATCGAGAACTCAAGCAACAGGCAGGTGACC      60
        ***** ***** ***** ***** ***** ** ** *** ***  ***** ** ** **

Mt      TATTCAAAGAGAAAGAATGGAATCCTTAAGAAAGCAAAGGAAATAAGTGTTCTTTGTGAT      120
Cb      TACTCAAAAAGAAGAAATGGGATCATGAAAAAAGCTAAGGAAATCAGTGTTCTTTGCGAG      120
Ca      TACTCAAAGAGAAAAACTGGGATCATGAAGAAGGCTAAGGAGATCACAGTTCTTTGTGAT      120
Pl      TACTCAAAGAGGAGGACTGGGATCTTGAAGAAGGCCACGGAGATAACTGTTCTCTGCGAT      120
Pd      TACTCTAAGAGGAAAAATGGGATTATAAAAAAAGCTAAGGAGATCACAGTTTTATGCGAT      120
Ck      TACTCAAAGAGAAGAAATGGGATCATGAAGAAGGCAAAGGAGATCACAGTTCTGTGTGAT      120
Vv      TACTCAAAGAGGAGAAATGGGATCATGAAGAAGGCCAAGGAGATCACTGTTCTCTGCGAT      120
        ** ** ** ** *  * *** **  * ** ** ** * *** ** *  *** * ** **

Mt      GCTCAAGTTTCCCTTATTCTCTTTGGTGCTTCTGGAAAGATGCATGAATACATCAGCCCT      180
Cb      GCTCACGTTTCTGTAGTCATCTTCGCTAGTTCTGGGAAGATGCATGAATTTTGCAGCCCA      180
Ca      GCTAAGGTTTCCCTCATCATCTTCGGTACTTCTGGAAAGATGCATGAATATATAAGTCCT      180
Pl      GCTCATGTTTCTCTTGTTATCTTTGCTACTTCAGGGAAAATGCACGAATACTGTAGCCCT      180
Pd      GCTCAAGTTTCTCTTGTCATCTTTGCTAGTTCTGGAAGGATGCATGAGTACTGCAGCCCT      180
Ck      GCTCATGTCTCTCTTGTAATCTTTGCTAGTTCTGGCAAGATGCATGAGTACTGCAGCCCT      180
Vv      GCTCATGTCTCTCTTGTTATCTTTGCTAGCTCTGGAAAGATGCACGAGTACTGTAGCCCT      180
        *** * ** **  *  *  **** * *   ** ** *  ***** ** *     ** **

Mt      TCTACCACGTTGATTGATATTCTGGACAGATACCAGAGAGCCTCTGGGAAAACCCTATGG      240
Cb      AATACTACGTTAGTTGATATGTTGGATCAGTACCACAAGTTGTCTGGGAAGAGGCTCTGG      240
Ca      TCAACAAATTTGGTTGAAATGTTGGATGCTTACCAGAGGTCTACTGGAAAGAAGCTGTGG      240
Pl      TCCACTACGGTGATTGATATTTTGGATAGATATCACAAGCAGTCTACAAAGAAGCTGTGG      240
Pd      TCCACTACGGTGGTCGATCTGTTGGACAAGTATCACAAGCAGTCTGGTAAGAGGCTATGG      240
Ck      TCAACTAAGTTGGTTGATATCTTGGATCGATATCACACACAGTCTGGGAAGAGGTTGTGG      240
Vv      TCTACAACGTTGATTGATATCTTGGATAGGTATCACAAGCAGTCTGGGAAGAGGCTCTGG      240
           ** *   *  * **  *  ****    ** ** *      **   ** *   * ***

Mt      GATGCTAAGCATGAGAACCTTAGCAATGAAATTGATAGAATCAAAAAGGAGAATGATAGC      300
Cb      GATGCAAAGCATGAGCATTTGGACAATGAAATCAACAGAATCAAGAAAGAGAATGACAGC      300
Ca      GATGCTAAGCATGAGAATTTAAGCAATGAAATTGATAGAGTTAAGAAAGAGAACGACAGC      300
Pl      GATCCTAAGCATGAAAATCTCAGCAATGAAGTGGATCGAGTCAAAAAAGAAAACGATAAC      300
Pd      GACGCTAAACATGAGAACCTCAGCAAAGAGATTGACAGAATCAAGAAAGAGAATGACAGC      300
Ck      GATGCAAAGCATGAGAATTTGAGCAATGAAATTGATAGAATCAAGAAGGAGAATGACAGC      300
Vv      GATGCAAAACATGAAAATCTCAGCAATGAATTGGATAGGATCAAAAAGGAGAATGATAGC      300
        **  * ** *****  *  *   *** **  *  *  *  * ** ** ** ** ** * *

Mt      ATGCAAATTGATCTCAGGCACTTGAAGGGAGAGGATATTACCTCACTGAATTACAAAGAG      360
Cb      ATGCAGATTGAGCTCAGGCACCTCAAGGGAGAAGATATCTCAACTTTGAACTACAAAGAG      360
Ca      ATGCAGATTGAGCTCAGGCACCTAAAGGGGGAAGACATCACATCTTTGAATTACAAAGAG      360
Pl      ATGCAGATCGAGCTCAGGCACCTCAAGGGAGAGGATGTTACTTCTCTGAGTATCAAAGAG      360
Pd      ATGCAGATTGAGCTGAGGCACCTGAAAGGGGAAGATATCTCATCATTGCACCACACAGAG      360
Ck      ATGCAGATTGAGCTCAGGCACCTGAAGGGGGAAGATATCACATCTTTGCACTACAAAGAG      360
Vv      ATGCAGATTGAACTCAGGCACCTGAAGGGGGAGGATATCTCATCTCTGCACCACAAAGAA      360
        ***** ** ** ** ****** * ** ** ** **  *  *  *  **      ** ***
```

Supplementary Figure S1. Alignment of partial cDNA sequences (360 nucleotides) of *PISTILLATA* genes from selected woody species and from the mode legume *Medicago truncatula*. The regions highlighted with red rectangles presented four mismatches among the sequences and these regions were selected for the design of degenerated oligos. Mt, *Medicago truncatula*; Cb, *Catalpa bungei*; Ca, *Coffea arabica*; Pl, *Paeonia lactiflora*; Pd, *Populus deltoides*; Ck, *Cornus kousa*; Vv, *Vitis vinífera.*

a

```
ATGGGTAGAGGCAAGATAGAGATCAAGAGAATAGAGAACTCGAGCAATAGGCAAGTGACA
 M  G  R  G  K  I  E  I  K  R  I  E  N  S  S  N  R  Q  V  T
TACTCCAAGAGGAGGAATGGGATCATGAAGAAGGCTAAGGAGATCACTGTACTTTGTGAT
 Y  S  K  R  R  N  G  I  M  K  K  A  K  E  I  T  V  L  C  D
GCTCAGGTCTCTCTTGTTATCTTTGGGAGTTCAGGCAAGATGCATGAGTACTGTAGCCCT
 A  Q  V  S  L  V  I  F  G  S  S  G  K  M  H  E  Y  C  S  P
TCCACTACGTTGACTGACATCTTGGATAAGTATCACAAGAAGTCTGGGAAGAGGTTGTGG
 S  T  T  L  T  D  I  L  D  K  Y  H  K  K  S  G  K  R  L  W
GATGCTAAACATGAGAACCTCAGCAACGAACTTGATAGAATCAAGAAAGAGAATGATCAT
 D  A  K  H  E  N  L  S  N  E  L  D  R  I  K  K  E  N  D  H
ATGCAGATTGAGCTGAGGCACTTGAAAGGAGAAGAAATAACATCTTTGAACCACAAAGAG
 M  Q  I  E  L  R  H  L  K  G  E  E  I  T  S  L  N  H  K  E
CTCATGACCATAGAGGAAGCCCTGGAAAATGGTCTTGCTTGTGTTCGCCACAAGCAGATG
 L  M  T  I  E  E  A  L  E  N  G  L  A  C  V  R  H  K  Q  M
GAATTCTTCACGATGATGCAGAAAAATGATAAGATGCTGGAGGACGAGAACAAGCAGCTC
 E  F  F  T  M  M  Q  K  N  D  K  M  L  E  D  E  N  K  Q  L
AATTTCGAGTTGCATCAGCAAGAGATCATTATGGGGAGTAGGGAGATGGAGAATGGATAT
 N  F  E  L  H  Q  Q  E  I  I  M  G  S  R  E  M  E  N  G  Y
CATCAGCGAGTGAGAGAGTATCCGCCCCAGATGCCCTTTGCCTTCCGCGTGCAGCCAATC
 H  Q  R  V  R  E  Y  P  P  Q  M  P  F  A  F  R  V  Q  P  I
CAGCCCAATCTGCAGGAGAGAATTTAG
 Q  P  N  L  Q  E  R  I  -
```

b

```
Seq569        CAGGAGAGAATTTAGAATAATAACATATAATTTTCTGGTATTAATTTGAACTCTAAACTA
Seq568        CAGGAGAGAATTTAGAATAATAACATATAATTTTCTGGTATTAATTTGAACTCTAAACTA
Seq570        CAGGAGAGAATTTAGAATAATAACATATAATTTTCTGGTATTAATTTGAACTCTAAACTA
Seq571        CAGGAGAGAATTTAGAATAATAACATATAATTTTCTGGTATTAATTTGAACTCTAAACTA
              ************************************************************

Seq569        AACTCAGACTCGGATTGTTTAATGTTAAGCCTTTTAGCTGTGATTGTTTGTCAAAAAAAA
Seq568        AACTCAGACTCGGATTGTTTAATGTTAAGCCTTTTAGCTGTGATTGTTTGTCATGGGGAC
Seq570        AACTCAGACTCGGATTGTTTAATGTTAAGCCTTTTAGCTGTGATTGTTTGTCATGGGGAC
Seq571        AACTCAGACTCGGATTGTTTAATGTTAAGCCTTTTAGCTGTGATTGTTTGTCATGGGGAC
              ****************************************************

Seq569        AAAAAAAAAAAAA
Seq568        GTCTTAGTTAAGCTTCTTCTAATAAGGTAAACTTGCTTTGTGAAACATTTATCGTGTGAT
Seq570        GTCTTAGTTAAGCTTCTTCTAATAAGGTAAACTTGCTTTGTGAAAAAAAAAAAAAAAAA
Seq571        GTCTTAGTTAAGCTTCTTCTAATAAGGTAAACTTGCTTTGTGAAAAAAAAAAAAAAAAAA


Seq569
Seq568        TAATGCTTGTCGTGGAAAAAAAAAAAAAAAAAAAAAAAAAAAAAAAAAAAAA
Seq570
Seq571        AAAA
```

Supplementary Figure S2. *PISTILLATA* gene sequence. (a) Coding sequence and protein from *PISTILLATA* gene from *Argania spinosa*. Conserved amino acid domains are highlighted in red (MADS domain), green (K-domain) and purple (PI-motif). Potential NLS sequences are underlined; (b) Sequences obtained by 3'RACE of the 3' untranslated region of the gene showing different lengths and polyadenylation position. The stop codon is marked in red.

```
AtPI          MGRGKIEIKRIENANNRVVTFSKRRNGLVKKAKEITVLCDAKVALIIFASNGKMIDYCCP    60
PhGLO2        MGRGKIEIKRIENSSNRQVTYSKRRNGIIKKAKEITVLCDAKVSLIIFGNSGKMHEYCSP    60
MtNGL9        MGRGKIEIKRIENSSNRQVTYSKRKSGILKKAKEINVLCDAQVSTIIIAPSGKMHEYISP    60
AsPI          MGRGKIEIKRIENSSNRQVTYSKRRNGIMKKAKEITVLCDAQVSLVIFGSSGKMHEYCSP    60
MtPI          MGRGKIEIKRIENSSNRQVTYSKRKNGILKKAKEISVLCDAQVSLILFGASGKMHEYISP    60
PhGLO1        MGRGKIEIKRIENSSNRQVTYSKRRNGILKKAKEISVLCDARVSVIIFASSGKMHEFSS-    59
AmGLO         MGRGKIEIKRIENSSNRQVTYSKRRNGIMKKAKEISVLCDAHVSVIIFASSGKMHEFCSP    60
              *************:.** **:***:.*::******.*****:*: :::. .*** :: .

AtPI          SMDLGAMLDQYQKLSGKKLWDAKHENLSNEIDRIKKENDSLQLELRHLKGEDIQSLNLKN    120
PhGLO2        STTLPDMLDGYQKTSGRRLWDAKHENLSNEIDRIKKENDNMQVKLRHLKGEDINSLNHKE    120
MtNGL9        STTLIDMLERYHKASGKRLWDAKHENLKNEIEKLKKENEDMQIQLRHLKGKDINTLNYKK    120
AsPI          STTLTDILDKYHKKSGKRLWDAKHENLSNELDRIKKENDHMQIELRHLKGEEITSLNHKE    120
MtPI          STTLIDILDRYQRASGKTLWDAKHENLSNEIDRIKKENDSMQIDLRHLKGEDITSLNYKE    120
PhGLO1        -TSLVDILDQYHKLTGRRLLDAKHENLDNEINKVKKDNDNMQIELRHLKGEDITSLNHRE    118
AmGLO         STTLVDMLDHYHKLSGKRLWDPKHEHLDNEINRVKKENDSMQIELRHLKGEDITTLNYKE    120
                 *  :*: *:: :*: * * ***:*.**::::**:*: :*:.******::* :** ::

AtPI          LMAVEHAIEHGLDKVRDHQMEILISKRRNEKMMAEEQRQLTFQLQQQEMAIASNARGMMM    180
PhGLO2        LMVLEEGLTNGLSSISAKQSEILRMVRKNDQILEEEHKQLQYALHQKEMAAMGGNMRMIE    180
MtNGL9        LMSLEDVLENGLLTVRHKQMEVYKMVKRNDKILKEENRELNFILQQQGYGSGRW---EI    177
AsPI          LMTIEEALENGLACVRHKQMEFFTMMQKNDKMLEDENKQLNFELHQQEIIMGSRE---ME    177
MtPI          LMALEESLENGLTGVRDKKMEVHRMFKRNGKILEDENKELNFLLQQHMALEGVGN---MH    177
PhGLO1        LMILEDALENGLTSIRNKQNEVLRMMRKKTQSMEEEQDQLNCQLRQLEIATMNRNMGEIG    178
AmGLO         LMVLEDALENGTSALKNKQMEFVRMMRKHNEMVEEENQSLQFKLRQMHLDPMNDNVMESQ    180
              ** :*. : :*   :  :: *.    ::: : : :*: .*   *:*

AtPI          R--------------DHDGQFGYRVQPIQPNLQEKIMSLVID   208
PhGLO2        EVYHQ----RDRDYEYQQMPFALRVQPMQPNLHERM------   212
MtNGL9        NGL---------------------------------------   180
AsPI          NGYHQ----RVREYP-PQMPFAFRVQPIQPNLQERI------   208
MtPI          GQWI--------------------------------------   181
PhGLO1        EVFQQR---ENHDYQ-NHMPFAFRVQPMQPNLQERL------   210
AmGLO         AVYDHHHHQNIADYE-AQMPFAFRVQPMQPNLQERF------   215
```

Supplementary Figure S3. Protein alignment of AsPI and functional characterized PI/GLO-related proteins from different species. Amino acid residues identical to all the sequences are indicated by stars (*). To improve the alignment, dashes were introduced into the sequence. The region designated as the PI motif is boxed. At, *Arabidopsis thaliana*; Ph, *Petunia hybrida*; Mt, *Medicago truncatula*; As, *Argania spinosa*; Am, *Antirrhinum majus.*

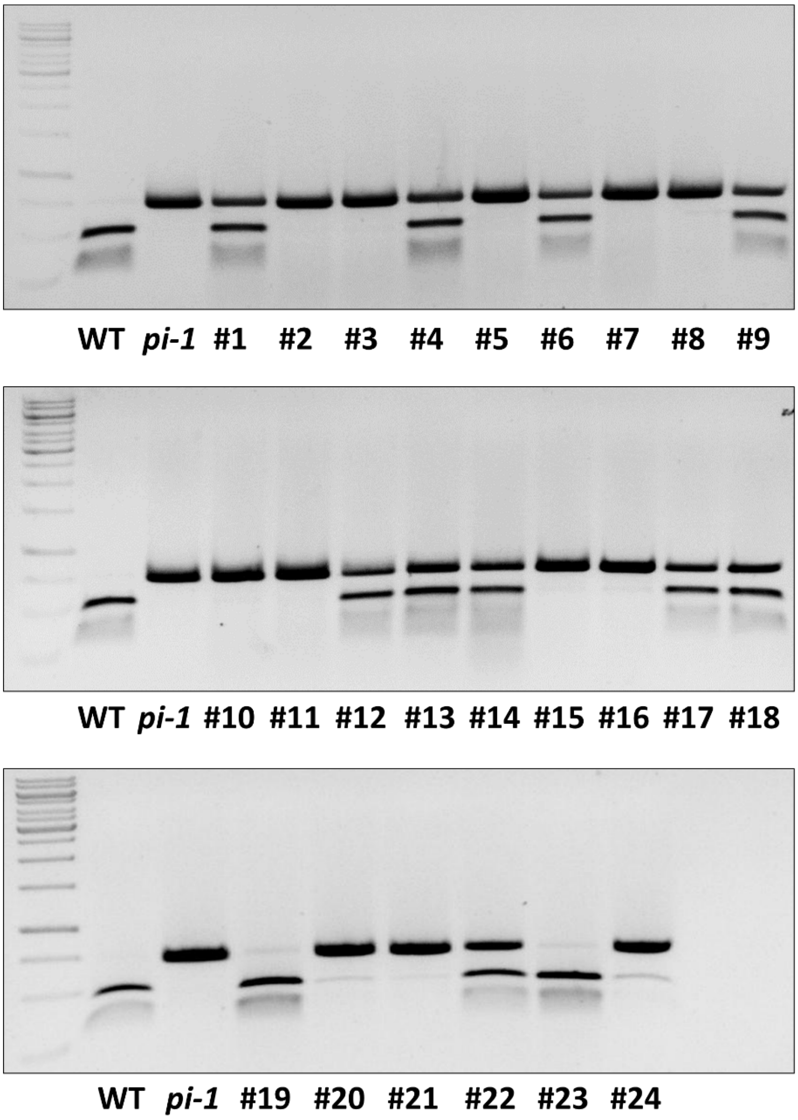

Supplementary Figure S4: Genotyping of transgenic plants transformed with 35S::*AsPI* for the presence of the *pi-1* mutation. A genomic PCR product (729 bp) was amplified and digested by *Bse*GI. Two fragments (451 and 278 bp) were obtained in the case of wild-type plants (lower bands are generally faint in the gels). *Bse*GI does not cut the 729 bp PCR fragment in *pi-1/pi-1* homozygous plants. Heterozygous *PI/pi-1* plants showed three bands. Twenty-four transformants were analysed.

Supplementary Table S1. Primers used in this work.

**Primer name**	**Primer sequences (5′ to 3′)**	
PI DEG FOR	ATG GG**D** AGA GG**H** AAG AT**W** GAG AT**M** AAG	Degenerated oligo
PI DEG REV	GTG CCT **S**AG **H**TC **R**AT **Y**TG CAT	Degenerated oligo
AsPI GSP2	GAG AAC CTC AGC AAC GAA CT	3' RACE
AsPI GSP7	CCT CTT CCC AGA CTT CTT GTG AT	5' RACE
PI argan ATG For	ATG GGT AGA GGC AAG ATA GAG	AsPI cDNA amplification
PI argan –STOP Rev	AAT TCT CTC CTG CAG ATT GGG C	
Q-AsPI For3	GGG ATC ATG AAG AAG CTA AGG	qRT-PCR
Q-AsPI Rev3	GGC TAC AGT ACT CAT GCA TCT T	
Arg YCF2 For	CCC GAA CCA AGG AAT CCC T	qRT-PCR
Arg YCF2 Rev	TCC CCT TCT TCA AAC TCC GAT	
AtTIP41 For	GTG AAA ACT GTT GGA GAG AAG CAA	qRT-PCR
AtTIP41 Rev	TCA ACT GGA TAC CCT TTC GCA	

Supplementary Table S2. Accession numbers of the *AP3/DEF*- and *PI/GLO*-related MADS-box genes used for phylogenetic analysis.

Gene	Species	GenBank Id
MtPI	*Medicago truncatula (Mt)*	FJ403468
PlPI	*Paeonia lactiflora (Pl)*	KC354379
CabuPI	*Catalpa bulguei (Cabu)*	KM112022.1
CaPI	*Coffea arabica (Ca)*	KJ483234
CorPI	*Cornus kousa (Cor)*	JQ753781
PdPI	*Populus deltoides (Pd)*	EU029172
VvPI	*Vitis vinifera (Vv)*	NM_001280946.1
MASAKO BP	*Rosa rugosa*	AB038462.1
SlTM6/FBP1	*Solanum lycopersicum (Sl)*	XM_004245154.4
PhGLO1	*Petunia Hybrida (Ph)*	M91190.1
PhGLO2	*Petunia Hybrida (Ph)*	X69947.1
AmGLO	*Antirrhinum majus (Am)*	AB516403
MtPI	*Medicago truncatula (Mt)*	FJ403468
MtNGL9	*Medicago truncatula (Mt)*	FJ403469
Paps PI-1	*Papaver somniferum (Ps)*	EF071994.1
Paps PI-2	*Papaver somniferum (Ps)*	EF071995.1
AtPI	*Arabidopsis thaliana (At)*	NM_122031
MtTM6	*Medicago truncatula (Mt)*	JN412097.1
MtNMH7	*Medicago truncatula (Mt)*	JN412096.1
AtAP3	*Arabidopsis thaliana (At)*	AY142590
PhTM6	*Petunia Hybrida (Ph)*	AF230704
PhDEF	*Petunia Hybrida (Ph)*	DQ539416.1
SlTAP3/TDR6	*Solanum lycopersicum (Sl)*	AY098734.2
AmDEF	*Antirrhinum majus (Am)*	HQ853386.1
MdMADS13	*Malus domestica (Md)*	NM_001328749.1
MdTM6	*Malus domestica (Md)*	AB081093.1
